# Submucosal Tunnel Endoscopic Resection for Esophageal Submucosal Tumors: A Multicenter Study

**DOI:** 10.1155/2018/2149564

**Published:** 2018-12-02

**Authors:** Sufang Tu, Silin Huang, Guohua Li, Xiaowei Tang, Haitao Qing, Qiaoping Gao, Jingwen Fu, Guoping Du, Wei Gong

**Affiliations:** ^1^Department of Gastroenterology, Nanfang Hospital, Southern Medical University, Guangzhou, China; ^2^Department of Gastroenterology, Shenzhen Hospital, Southern Medical University, Shenzhen, China; ^3^Department of Gastroenterology, Shunde Hospital, Southern Medical University, Foshan, China; ^4^Department of Gastroenterology, Affiliated Hospital of Southwest Medical University, Luzhou, China

## Abstract

**Background:**

Submucosal tumors (SMTs) are primarily benign tumors, but some may have a malignant potential. Endoscopic submucosal dissection that has been used for removing esophageal SMTs could cause perforation. Submucosal tunnel endoscopic resection (STER) is an improved and an effective technique for treating esophageal SMTs.

**Aims:**

This study was conducted to evaluate the efficacy and safety of STER for treating esophageal SMTs.

**Methods:**

A retrospective study design was adopted to analyze the baseline characteristics, clinical outcomes, and follow-up data of patients with esophageal SMTs, which originated from the muscularis propria layer and were treated with STER from September 2011 to May 2018.

**Results:**

A total of 119 lesions were included from 115 patients who were successfully treated with STER. The mean age of the patients was 49.7 ± 10.7 years. The lesions were primarily located in the middle and lower esophagus. The mean size of the lesions was 19.4 ± 10.0 mm. The mean operation duration was 46.7 ± 25.6 min, and the mean duration of hospitalization was 5.9 ± 2.8 days. The total en bloc resection rate and the complete resection rate were 97.5% and 100%, respectively. Regarding complications, there were 9 (7.8%) cases of perforation, 2 (1.7%) cases of pneumothorax, and 9 (7.8%) cases of subcutaneous emphysema. Histopathological results revealed 113 (95.0%) cases of leiomyoma, 5 (4.2%) cases of gastrointestinal stromal tumors, and 1 (0.8%) case of a granular cell tumor. During the mean 15-month follow-up, there were no cases of recurrence and distant metastasis.

**Conclusions:**

STER is a safe and feasible technique for treating esophageal SMTs originating from the muscularis propria layer.

## 1. Introduction

Submucosal tumor (SMT) is a rare esophageal disease, comprising <1.0% among esophageal tumors [1] and generally does not manifest clinical symptoms; because of this, the detection rate is low. Esophageal SMTs are primarily benign, although some SMTs may possess the biological characteristics of malignant tumors. Thus, a complete resection needs to be performed to obtain the pathological diagnosis [2–4]. Surgery has been the primary treatment for esophageal SMTs [5, 6]; however, it is more traumatic for local tissues [7]. Furthermore, it is often difficult to identify SMTs protruding into the lumen without the assistance of a gastroscope during the procedure. With the development of the endoscopic technique, endoscopic submucosal dissection (ESD) has been gradually used for treating esophageal SMTs [4, 8]. However, the primary complication associated with ESD is that it would easily cause perforation because the tumors originating from the muscularis propria (MP) layer need full-thickness resection. Submucosal tunnel endoscopic resection (STER) is an improved endoscopic technique based on ESD and has been gradually applied for treating esophageal SMTs [9, 10]. The purpose of this study was to evaluate the efficacy and safety of STER for treating esophageal SMTs.

## 2. Materials and Methods

### 2.1. Clinical Data

This study conducted a retrospective analysis of the data of 119 lesions from 115 patients with SMTs originating from the MP layer, who underwent STER between September 2011 and May 2018 at the Digestive Department of Nanfang Hospital, Shenzhen Hospital, and Shunde Hospital of Southern Medical University. Indications included (1) endoscopy showing esophageal SMTs, excluding malignant tumors, (2) EUS examination showing that the tumor originated from the MP layer and protruded into the lumen, and (3) patients who could tolerate anesthesia with tracheal intubation.

All patients underwent preoperative examination, including ECG, chest X-ray, routine blood test, and blood coagulation test. A CT scan was also performed to exclude the possibility of malignant tumors and distant metastasis. All patients were informed of the procedure and received detailed explanations about the treatment and complications, and informed consent was obtained before performing the STER procedures. All the operations were performed by endoscopy doctors experienced in endoscopy therapy. This study was approved by the ethics committee of the Nanfang Hospital, Southern Medical University (Guangzhou, China).

### 2.2. Endoscopic Equipment and STER Procedures

The endoscopic equipment primarily included an endoscope (GIF-Q260J; Olympus, Tokyo, Japan), a transparent distal cap (MH-588; Olympus), a high-frequency electrogenerator (VIO200D; Erbe, Germany), a carbon dioxide (CO_2_) insufflator (UCR; Olympus, Japan), and a snare (SD-210U-25; Olympus, Japan). A hybrid knife (Type-I; Erbe, Tübingen, Germany) was used to resect the tumors completely. A hemostatic forceps (Microclip; Olympus) was used to control bleeding, while endoscopic clips (Micro-Tech; Nanjing, China) were used for closure of the wound.

**Figure 1:**
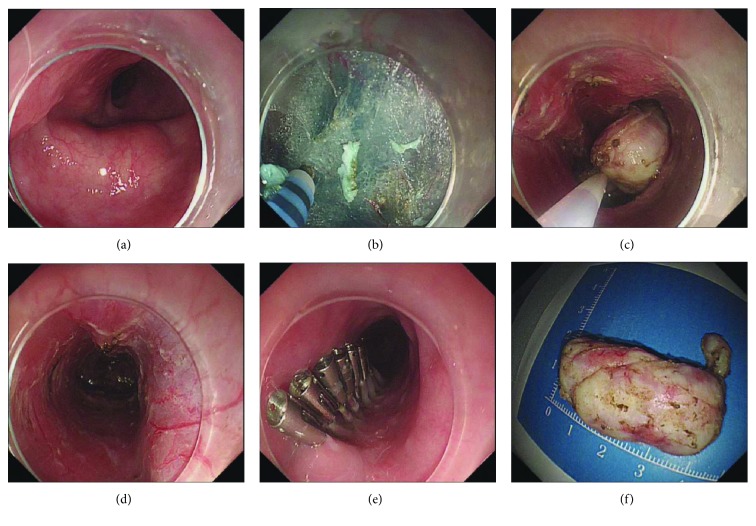
Case illustration of submucosal tunnel endoscopic resection for esophageal submucosal tumor (SMT). (a) A SMT located at the midesophagus shown by white light. (b) A 2 cm longitudinal mucosal incision was made using a hybrid knife, approximately 5 cm proximal to the SMT, and a straight submucosal tunnel was made until the tumor was visible. (c) Resection was done along the margin of the SMT using the hybrid knife. (d) The wound of the submucosal tunnel was checked after the removal of the tumor. (e) Metal clips were used to close the entrance of the tunnel. (f) The resected specimen was measured, and the final pathological diagnosis confirmed a 35 mm leiomyoma.

All patients had fasted for 8 h before the operation and underwent STER under general anesthesia with tracheal intubation. The STER procedures are described below (Figure 1).

#### 2.2.1. Creation of the Tunnel Entrance

A mixture of 10 ml saline and 0.2% indigo carmine was injected 5 cm proximal to the SMT. When the mucosa was fully lifted, a 2 cm longitudinal mucosal incision was made on the mucosal layer and the submucosal layer was exposed to create a tunnel entrance.

#### 2.2.2. Creation of the Submucosal Tunnel

A transparent cap was attached to the front of the endoscope; the mucosa was separated from the muscular layer, and then, a straight submucosal tunnel was established using a hybrid knife. The submucosal layer was gradually dissected until the tumor was exposed to the endoscopic view. Continuous dissection was performed until the tunnel was created 2 cm distal to the tumor. Timely electrocoagulation was used to stop bleeding during the operation.

#### 2.2.3. Dissection of the SMTs

When the tumor was fully exposed, a hybrid knife was used to gradually dissect the tumor along the tumor capsule, until it was completely resected, and then the specimen was removed.

#### 2.2.4. Examination of the Tunnel Incision

The wound in the tunnel was flushed, and electrocoagulation was performed to prevent bleeding.

#### 2.2.5. Closure of the Tunnel

The tunnel was gradually closed by using metal clips to make a continuous chain suture from the bottom up.

### 2.3. Postoperative Clinical Management

All the patients were required to remain on nil per os for at least 24 h before resuming their diet and water after the operation. To prevent postoperative infection, patients were administered routine prophylactic antibiotics for 48 h as needed, which primarily included the first- or second-generation cephalosporin drug. Moreover, they must at least receive proton pump inhibitors for 3 days, and their vital signs were closely monitored to observe the occurrence of complications. Postoperative complications primarily included subcutaneous emphysema, pneumothorax, pulmonary infection, and hemorrhage.

Perforations could be identified by endoscopy or by the discovery of free gas in the X-rays or the CT scan. Therefore, when a perforation was detected, it was important to perform an endoscopic suture, extend the duration of fasting and water deprivation and the use of antibiotics, and provide gastrointestinal decompression if necessary. If the patients suffered from progressive dysphagia after the operation, it was necessary to consider the possibility of internal bleeding in the tunnel. Therefore, the use of coagulation forceps during the endoscopy was important to stop the bleeding. Additional surgery was necessary for uncontrolled perforations and bleeding.

All patients were recommended to be followed up with gastroscopy or endoscopic ultrasonography for 6 and 12 months after the operation. If there were no residual tumors or recurrences, the patients could be followed up by an endoscopic examination once a year.

### 2.4. Statistical Analysis

Data were analyzed through descriptive statistics. Quantitative data were expressed by mean (±standard deviation) or median (range). Qualitative data were expressed as *n* (%). All data were statistically analyzed using the standard statistical software SPSS 19.0 (IBM).

## 3. Results

### 3.1. Baseline Characteristics of Patients

The baseline characteristics of patients are shown in Table 1. A total of 119 SMTs from 115 patients were resected by STER. The mean age of the patients was 49.7 ± 10.7 years (range: 26–71 years). The ratio of female to male was 39 : 76. The lesions were primarily located in the middle and lower esophagus. In total, 10 (8.4%) lesions were located in the upper esophagus (<23 cm from the incisors), 58 (48.7%) lesions were located in the median esophagus (23–32 cm from the incisors), and 51 (42.9%) lesions were located in the lower esophagus (32 cm from the incisors to the gastroesophageal junction). EUS examination showed that all the tumors originated from the MP layer. The mean size of the lesions was 19.4 ± 10.0 mm (range: 8–60 mm). The size of the majority of tumors was <30 mm. There were 110 (92.4%) tumors measuring <30 mm, 5 (4.2%) measuring between 30 and 40 mm, and 4 (3.4%) measuring >40 mm.

### 3.2. Clinical Outcomes of STER

All patients were successfully treated with the STER procedure. All the esophageal SMTs originated from the MP layer, 87 of which were located in the superficial MP layer, while the remaining 32 were located in the deep MP layer. As shown in Table 2, the mean duration of operation was 46.7 ± 25.6 min (range: 10–150 min). In the early period, air was used in 4 (3.5%) patients during the STER operation, whereas the subsequent 111 (96.5%) patients were insufflated with CO_2_. All tumors were successfully resected by STER. The en bloc rate of tumors was 97.5%, whereas the complete resection rate was 100%. There were three cases of tumors with a long diameter of >40 mm and having a lobulated appearance. However, although the three cases were completely resected, the tumors were too large to be removed out of the tunnel, and these three tumors were finally removed in pieces. After the operation, most of the patients recovered well with no occurrence of postoperative complications. However, because the tumors were relatively large and located in the deep MP layer, the longitudinal muscle was cut off during the operation to dissect the tumors completely. Therefore, 9 (7.8%) cases of esophageal wall perforation occurred during the operation. After clamping the tunnel entrance using metal clips, there were no cases of delayed perforation and digestive leakage. Two (1.7%) patients had pneumothorax during the STER operation, but they recovered well after receiving closed thoracic drainage for 1 week. In addition, 9 (7.8%) patients had subcutaneous emphysema in the neck and chest area, but it disappeared soon spontaneously during postoperative observation. No other complications were found during postoperative follow-up. The mean hospitalization duration was 5.9 ± 2.8 days (range: 3–15 days).

### 3.3. Histopathology and Follow-Up Results

The histopathological results revealed 113 (95.0%) cases of leiomyoma, 5 (4.2%) cases of gastrointestinal stromal tumors (GISTs), and 1 (0.8%) case of a granular cell tumor. Based on the modified National Institutes of Health (NIH) classification suggested by Joensuu, the GISTs were graded according to the tumor size, tumor location, and the number of mitosis per 50 high power fields. Five cases diagnosed as GISTs were at low or extremely low risk. During a median follow-up of 15 months (range: 1–71 months), there were no tumor recurrences or distant metastasis. Moreover, there were no cases of delayed perforation or bleeding, digestive tract leakage, or other serious complications.

## 4. Discussion

Upper gastrointestinal SMTs are a type of tumors that are rarely encountered and generally an incidental finding in daily clinical practice owing to their nature of rarely causing clinical symptoms. The development of the endoscopic technique has significantly improved the detection rate of esophageal SMTs. Open surgery and thoracoscopic surgery have long been considered as standard methods for treating upper gastrointestinal SMTs [5, 6, 11]. However, endoscopic resection has been gradually applied to treat esophageal SMTs in recent years. Endoscopic therapies, including ESD, endoscopic submucosal excavation, and endoscopic full-thickness resection (EFR), can be used to remove SMTs successfully [12, 13]. Nevertheless, although ESD and EFR have been recognized as quick, effective, and minimally invasive methods for removing SMTs, complications such as perforation occur frequently [14, 15], which are difficult to manage using endoscopic treatment methods and might even require surgical intervention. Consequently, several endoscopic physicians began attempting the use of STER to resect esophageal SMTs.

Xu et al. of Shanghai Zhongshan Hospital first reported about the use of STER technique to treat upper gastrointestinal SMTs [9]. Subsequently, our center reported about the use of STER to treat esophageal and cardiac SMTs [16]. An increasing number of researchers from China and across the world have successively explored the STER technique [17–20]. The safety and efficacy of STER for treating esophageal SMTs have been verified by a large number of clinical studies [21, 22].

In the present study, we included 119 cases of SMTs from 115 patients who had been identified through endoscopic examination during a period of 7 years. All tumors were successfully removed. The mean operation duration was 46.7 min, which was found to be consistent with other studies that reported a range of 40–78.3 min. Domestic experts have reached consensus on endoscopic diagnosis management of gastrointestinal SMT [23]. Then, combining the literature review with our own experience [24–26], we believe that patients who underwent STER should meet the following conditions: (1) the tumor being <4 cm, (2) the tumor originating from the MP layer and protruding into the lumen, and (3) the tumor being confirmed to be benign or excluding the possibility of malignancy. Before the operation, we required all the patients to receive a clinical evaluation. Moreover, EUS and CT scan were necessary to determine the characteristics and growth pattern of the tumors. EUS can determine the origin and nature of tumors, whereas CT scan can identify peripheral vessels, lymph nodes, and distant metastasis.

The diameter of the 115 lesions, for which we chose to perform STER, was evaluated to be <4 cm by a common endoscope and EUS. We believe that it is difficult to dissect tumors measuring >4 cm because of the limited operating space within the tunnel, which makes it difficult to remove the tumor from the tunnel. In this study, we found four patients in our center with tumors measuring >4 cm as measured by EUS. The patients were fully informed of the risk of failure with the difficult procedure performed under the guidance of an endoscope and the possibility of surgical treatment; however, the patients still demanded endoscopic resection and signed the informed consents. Based on the general operation procedure, the tunnel was successfully constructed 2 cm distal to the tumor. The mucosa was incised longitudinally, and the incision length was about 3 cm to achieve tunnel decompression. Continuous dissection of the tumor was performed until the tumor was successfully removed out of the tunnel. The tunnel space became larger, and the tumors could be resected and removed easily. Endoscopic tunnel decompression is a new attempt for removing SMTs measuring >4 cm and was successfully used in one case. However, although the other three cases were also completely resected, the tumors were too large to be removed out of the tunnel. Therefore, these three tumors were cut into pieces using a snare. The fragmentation excision method might lead to the possibility of tumor recurrence, although there is no such report in the case of endoscopic tunnel treatment for upper gastrointestinal tumors. Moreover, these three patients were monitored more closely. Surveillance endoscopies were performed every 3–6 months over the first year postprocedure and then annually thereafter. Eventually, the pathological results revealed that all these four tumors were leiomyoma and there was no recurrence in these four patients during the 3-year follow-up.

We found multiple SMTs in three patients, all located in the adjacent esophagus. We successfully removed the tumor in a tunnel by establishing a long tunnel. There were no complications during and after the operation. Some researchers have also reported the possibility of using STER procedure to remove multiple SMTs in the upper digestive tract. Chen et al. reported the successful removal of SMTs in the esophagus and cardia in the meantime using STER [27]. Zhang et al. reported that 49 SMTs in 23 patients were successfully resected using the STER technique [28]. The complications were successfully controlled by conservative medical management after the operation. As a result, combined with our own experience and the literature reports, we suggest that the STER procedure can be used to remove multiple adjacent SMTs in one operation. Using this method, mucosal damage and other complications can be avoided for repeatedly establishing the tunnel.

The incidence of perforation in our three centers was 7.8% (9/115) in terms of intraoperative complications. All the lesions were quickly excised, and the tunnel entry was closed by metal clips. Intraoperative complications such as pneumothorax and subcutaneous emphysema occurred in the patients, but no surgical treatment was required after the treatment. During the operation, there were two patients with subcutaneous emphysema and pneumothorax. Another seven patients showed pure subcutaneous emphysema. Several researchers have also reported that STER was associated with a higher incidence of subcutaneous emphysema, but after closing the tunnel by the metal clips, no special treatment was required. There were nine cases of gas-related complications in our centers, three of which were air injection and six occurred during CO_2_ insufflation. CO_2_ has been fully confirmed to be a safe gas that can be rapidly absorbed by the body. CO_2_ cannot only significantly reduce postoperative pain and shorten the postoperative recovery time but can also effectively reduce the incidence of pneumomediastinum and air embolism [29]. Therefore, we recommend that CO_2_ insufflation be used routinely while performing STER.

After taking out the tumor, the wound was rinsed and observed carefully and electrocoagulation was applied to suspected bleeding spots. There was no delayed bleeding in all the patients after the operation. All the specimens were sent for pathological examination, and the results revealed that 113 cases were leiomyoma, 5 cases were GISTs, and 1 case was a granular cell tumor. Although there are no guidelines recommending endoscopic resection for treating GISTs, several studies have demonstrated that endoscopic resection was safe and effective for removing small GISTs. Small GISTs primarily have a low or extremely low risk. Joo et al. [30] conducted a comparative study on endoscopic versus surgical resection of GIST in the upper GI tract and evaluated the long-term follow-up results. During 45.5 months of follow-up, the recurrence rate was not significantly different between the 2 groups. Park [31] reported clinical results and long-term follow-up outcomes and found that the recurrence rate was relatively low (2.2%) during 46 months of follow-up and suggested that endoscopic resection is a feasible and effective alternative therapeutic modality for lower risk GISTs. What is more, Chinese consensus on endoscopic diagnosis management of gastrointestinal SMTs [23] has pointed out that STER can be used for esophageal SMTs, including benign leiomyoma and some low-risk GISTs. However, preoperative evaluation, such as EUS and CT scan, and postoperative long-term follow-up are necessary for esophageal SMTs. In this study, all patients received EUS and CT evaluation before the operation and these five cases proved to be no recurrence and metastasis during follow-up period.

Compared with surgical intervention and other endoscopic resection methods, the STER technique had the following obvious advantages: (1) By establishing a submucosal tunnel, SMTs can be resected and the blood vessels of the wound can be treated under direct vision. (2) According to literature reports, ESD therapy was associated with a perforation rate of 6.1%–15% for SMTs originating from the MP layer, and several large metal clips or suture devices were needed for closing the wound, which might cause a gastrointestinal fistula. Tunnel technology can guarantee the integrity of the tumor mucosal layer, and thus, the integrity of the gastrointestinal mucosa could be maintained. (3) Only a few small metal clips are needed to close the tunnel entry; thus, the STER technique was found to be simple and practicable.

## 5. Conclusions

STER is a safe and feasible technique for treating esophageal SMTs originating from the MP layer. However, our study still had some limitations. First, the number of cases enrolled in our study was not adequate and the follow-up time was too short. Furthermore, we performed pieces' resection for three large tumors, which may increase the risk of recurrence and metastasis. Finally, we successfully treated large esophageal SMTs using “tunnel decompression” for the first time; however, this was an attempt with only a few cases and more cases are needed to explore whether the technique is feasible. Future studies must enroll a larger number of patients with a longer follow-up period to confirm the long-term outcome. In addition, multicenter randomized controlled studies should be conducted to prove the efficacy and safety of STER.

## Figures and Tables

**Table 1:** Baseline characteristics of patients.

	*N* (%)
No. of patients	115
No. of lesions	119
Age, yr (range)	49.7 ± 10.7 (26–71)
Sex (female/male)	39/76
Tumor location	
Upper esophagus	10 (8.4%)
Median esophagus	58 (48.7%)
Lower esophagus	51 (42.9%)
Tumor distribution	
Superficial MP	87 (73.1%)
Deep MP	32 (26.9%)
Tumor size, mm (range)	19.4 ± 10.0 (8–60)
No. of tumors of different sizes, *n* (%)	
Φ ≤ 30 mm	110 (92.4%)
30 < Φ ≤ 40 mm	5 (4.2%)
Φ > 40 mm	4 (3.4%)

**Table 2:** Clinical and pathological outcomes of STER in patients with SMTs.

	Overall
Operation time, min (range)	46.7 ± 25.6 (10–150)
Insufflation, *n* (%)	
Air	4 (3.5)
CO_2_	111 (96.5)
En bloc resection, *n* (%)	116 (97.5)
Complete resection, *n* (%)	119 (100)
Complication, *n* (%)	
Perforation	9 (7.8)
Pneumothorax	2 (1.7)
Subcutaneous emphysema	9 (7.8)
Pneumoperitoneum	0 (0)
Delayed bleeding	0 (0)
Delayed perforation	0 (0)
Pathological diagnosis, *n* (%)	
Leiomyoma	113 (95.0)
Gastrointestinal stromal tumor	5 (4.2)
Granular cell tumor	1 (0.8)
Hospitalization time, days (range)	5.9 ± 2.8 (3–15)
Follow-up time, months (range)	15 (1–71)
Recurrence rate (%)	0

## Data Availability

The data used to support the findings of this study are available from the corresponding author upon request.
